# Pregnancy-associated malaria and malaria in infants: an old problem with present consequences

**DOI:** 10.1186/1475-2875-13-271

**Published:** 2014-07-11

**Authors:** Violeta Moya-Alvarez, Rosa Abellana, Michel Cot

**Affiliations:** 1 Institut de Recherche pour le Développement, UMR 216 Mère et enfant face aux infections tropicales, Faculté de Pharmacie Paris Descartes, 4 Avenue de l’Observatoire, 75270 Paris, France; 2 Université Pierre et Marie Curie (Paris 6), Centre Biomédical des Cordeliers, 15, rue de l’Ecole de Médecine, 75006 Paris, France; 3 Réseau doctoral de l’Ecole des Hautes Etudes en Santé Publique, Avenue du Professeur Léon-Bernard, CS 74312-35043 Rennes, France; 4 Departament de Salut Pública, Facultat de Medicina, Casanova 143, 08036 Barcelona, Spain

**Keywords:** Pregnancy-associated malaria, Immune tolerance, Intermittent preventive treatment in pregnancy, Parasitaemia, Infancy, Sulphadoxine-pyrimethamine

## Abstract

Albeit pregnancy-associated malaria (PAM) poses a potential risk for over 125 million women each year, an accurate review assessing the impact on malaria in infants has yet to be conducted. In addition to an effect on low birth weight (LBW) and prematurity, PAM determines foetal exposure to *Plasmodium falciparum in utero* and is correlated to congenital malaria and early development of clinical episodes during infancy. This interaction plausibly results from an ongoing immune tolerance process to antigens *in utero*, however, a complete explanation of this immune process remains a question for further research, as does the precise role of protective maternal antibodies. Preventive interventions against PAM modify foetal exposure to *P. falciparum in utero*, and have thus an effect on perinatal malaria outcomes*.* Effective intermittent preventive treatment in pregnancy (IPTp) diminishes placental malaria (PM) and its subsequent malaria-associated morbidity. However, emerging resistance to sulphadoxine-pyrimethamine (SP) is currently hindering the efficacy of IPTp regimes and the efficacy of alternative strategies, such as intermittent screening and treatment (IST), has not been accurately evaluated in different transmission settings. Due to the increased risk of clinical malaria for offspring of malaria infected mothers, PAM preventive interventions should ideally start during the preconceptual period. Innovative research examining the effect of PAM on the neurocognitive development of the infant, as well as examining the potential influence of HLA-G polymorphisms on malaria symptoms, is urged to contribute to a better understanding of PAM and infant health.

## Background

Pregnancy-associated malaria (PAM), defined as peripheral or placental infection by *Plasmodium,* presents as a major public health concern due to significant adverse health effects on both the mother and the foetus. Women are increasingly susceptible to malaria infection during pregnancy since *Plasmodium falciparum*, the most common parasite responsible for malaria, avoids spleen clearance through expression of proteins that bind to the chondroitin sulphate A (CSA) in the placental intervillous space [1-3]. Consequently, the foetus is initially exposed to malaria *in utero*. Epidemiological studies estimate 125 million pregnancies are at risk of malaria infection every year [4] within a purposed estimate of 32 million women who become pregnant every year in malaria endemic sub-Saharan Africa countries [5].

The effects of pregnancy-associated malaria on infants include stillbirth, congenital malaria, foetal anaemia, and low birth weight (LBW), caused by intra-uterine growth retardation (IUGR) and pre-term delivery [6-11]; considering the subsequent adverse health outcomes PAM related deaths would account for 75,000 to 200,000 infant deaths in sub-Saharan Africa [12].

Ultimately, pregnancy-associated malaria determines foetal exposure to *P. falciparum in utero.* Indeed, placental malaria is identified as a significant indicator for increased susceptibility to malaria during infancy [13-18]. In turn, PAM control strategies modify foetal exposure to *P. falciparum in utero*.

The prevalence of PAM is influenced by transmission, the immunity of the mother, and protective measures, such as insecticide-treated nets (ITNs) or intermittent preventive treatment in pregnancy (IPTp) [10]. Despite the considerable literature on PAM epidemiological and clinical outcomes, no clear conclusions regarding PAM’s effect on malaria in infants have been accurately reported. Further well-known risk factors for malaria in infants include high transmission or HIV co-morbidity [19], but exploring PAM influence on malaria risk during infancy could significantly contribute to better understand *Plasmodium* infection among infants. This review aims to revisit the present evidence on pregnancy-related factors that influence malaria in infants, including the effect of control interventions and novel research perspectives. Results are presented by the following topic areas: the epidemiological evidence on the effect of PAM on malaria in the offspring, the risk factors determining exposure to *Plasmodium in utero,* with special regard to control interventions, such as IPTp and ITNs, and the influence of the increasing resistance to SP-IPTp on malaria perinatal outcomes. Finally, new research perspectives to examine the effect of PAM on infant health are discussed.

## Methods: search strategy and selection criteria

A systematic literature specifying the epidemiology of malaria in infants with a focus on malaria risk factors in infants, was realized between the 10th January 2012 and the 9th June 2014 utilizing PubMed, the Cochrane Library, Global Health and World Health Organization regional databases. In total, 1,136 articles in English, French, Spanish and Portuguese were classified for review. A combination of standardized terms were used as search criteria; concerning PubMed, the search terms utilized were the Medical Subjects Headings (MeSH) “Parasitaemia” OR “Malaria” OR “Anaemia”. In addition, complementary articles, reports, and studies were identified through review and citations. Search criteria for relevant PAM and IPTp studies accepted all designs with the sole caveat that they originated from a malaria endemic country. Three-hundred and fifty-five articles were selected for final review. Due to the limited number of studies and reviews, no sensitivity analysis was realized. No date restrictions were applied and publication bias was not addressed.

### Pregnancy-associated malaria and malaria in infants: epidemiological evidence

Pregnancy-associated malaria is consistently associated with an increased malaria risk during infancy [13-16,18] and has been associated with congenital malaria, increased malaria episodes, anaemia, and non-malaria fever episodes in infants [10,20]. The principal findings of several reviewed studies are presented in Table 1.

**Table 1:** Influence of maternal parasitemia on malaria in infants

**Cohort**	**Study design and simple size**	**Time period**	**Transmission setting**	**Malaria prevention strategy during pregnancy**	**Treatment drug regime**	**Proportion of maternal peripheral parasitemia at delivery**	**Proportion of placental parasitemia**	**Proportion of neonatal parasitemia**	**Infant follow-up period**	**Median time to first parasitemia (days, min, max)**	**Association of infant malaria with PAM**	**Early infant parasitemia <3 months**
Mangochi [21] (Malawi)	Clinical trial on comparative efficacy of CQ or MQ; infant cohort follow-up (1766 women at delivery and 1289 infants)	1988-1990	Perennial with seasonal peaks	CQ and MQ	CQ	CQ: 20.3% MQ: 4.1%	CQ: 25.1% MQ: 6.2%	CQ: 8.6% MQ: 3.1%	12 months	199 (192-207)	at 3 months: 1.1 (0.7-1.9)	18.5%
Ebolowa [13] (Cameroon)	Infant cohort follow-up (197)	1993-1995	Perennial with seasonal peaks	CQ	CQ		22.84% (Primigravid: 69%; Multigravid: 31%)		24 months	PM+: 217; PM-:350	at 6 months: PM+: 36%; PM-: 14%, p<0.05 at 2 years: PM+: 46.5%; PM-: 38.5%, p=0.6	≈12%
Muheza [14] (Tanzania)	Infant cohort follow-up (453)	2002-2004	Perennial with seasonal peaks (400 infective mosquito bites each year)	SP (area with 68% resistance 14-day treatment failure rate)			15.2% (Primigravid≤2: 24%; Multigravid>2: 5.6%)		12 months	266 (238–294) PM-:273 (245-322) PM+: 244 (147-266);	Primigravidae: PM+:AOR= 0.21, (0.09–0.47) PM-: Reference*** Multigravidae: PM+: AOR =1.59, (1.16–2.17) PM-:AOR=0.67, (0.50–0.91)	PM+ ≈20%; PM-≈10%
Lambarené [15] (Gabon)	Infant cohort follow-up (527)	2002-2004	Perennial	No		10.5%*	9.48%		30 months	Primigravidae: PM+:107 (83-139) PM-:102 (29-205) Multigravidae: PM+:111 (13-189) PM-:92 (27-208)	PM+:AOR= 2.1, (1.2–3) PM-: Reference**	PM+ ≈2%; PM-≈0%
Manhiça [22] (Mozambique)	Clinical trial on the efficacy of SP compared to placebo; infant cohort follow-up (1030 women at delivery and 997 infants)	2003-2005	Perennial with seasonal peaks	ITNs vs ITNs+SP	SP-AQ	ITNs+ placebo:15.15% ITNs+SP: 7.1%	ITNs+ placebo:52.27% ITNs+SP: 52.11%	ITNs+ placebo:1.15% ITNs+SP: 0.92%	12 months		Clinical PAM: AOR=1.96 (1.13–3.41) Acute PM: AOR= 4.63 (2.1-10.24) Chronic PM: AOR=3.95 (2.07-7.55) PM-: Reference	
Tori Bossito [17,23] (Benin)	Infant cohort follow-up (550)	2007-2008	Perennial with seasonal peaks (400 infective mosquito bites each year)	SP	AL		11%	0.83%	12 months	PM+: 34 (4-83); PM-: 43 (4-85)	ITN:AOR=2.13 (1.24–3.67) No ITN: AOR=1.18 (0.60–2.33)	20.3%
Mono [24] (Benin)	Mother and infant cohort follow-up (218)	2008-2010	Mesoendemic (1-35 bites/person/year)	SP	Quinine or SP		3.67%		12 months	PAM+: 362 (18-390) PAM-: 365 (64-449)	PAM during the 3rd trimester of pregnancy: AOR= 4.6 (1.7; 12.5) PAM during the 1st and 2nd trimesters non significant	

PM: Placental malaria, PAM: Pregnancy associated malaria and AOR: Adjusted Odds Ratio.

*data from a reference article.

**the association between placental malaria and malaria in the child was only statistically significant for children who were randomized to receive the sulphadoxine-pyrimethamine intervention (AHR=3 (1.5-6)).

***Analysis of the effect of IPTp on parasitemia of the offspring was performed for 882 women of this cohort. Among them, 21.6% received no IPTp, 42% one dose, and 36.4% two or more doses.

Congenital malaria, defined as the presence of asexual *P. falciparum* parasites in the cord blood or in the peripheral blood during the first week of life [25], is the result of transplacental transmission of parasites just before or during delivery. Congenital malaria rates range between 0.83 and 5.96% [17,25-29] in recent epidemiological studies. The introduction of molecular techniques has increased the detection of cord blood parasitaemia raising prevalence rates to 33% [30]. Congenital malaria might entail clinically relevant symptoms in some cases, such as high fever and convulsions, anaemia, hepatosplenomegaly, jaundice, anorexia, vomiting, diarrhoea, drowsiness, pallor, respiratory distress, and cyanosis [30,31]. Although congenital malaria is an important factor in the differential diagnostic of neonatal fever in endemic countries, severe symptoms are rare and, hence, it does not appear to constitute an epidemic at present.

PAM is also associated with earlier episodes, as well as overall clinical malaria episodes, in infants [13-15,17,23]. In a landmark longitudinal cohort study of infants in Cameroon, placental *P. falciparum* infection was associated with infant malaria between four and six months, and parasitaemia rates were higher between five to eight months in offspring of placenta-infected mothers compared to offspring of mothers without placental infection independently of congenital infection [13] (at 6 months: PM+: 36%; PM-: 14%, p < 0.05). A study in Tanzania found an interaction between gravidity and placental malaria. The findings demonstrated that the offspring of multigravid women with placental malaria had the highest odds of subsequent malaria episodes (Adjusted Odds Ratio (AOR = 1.59) 95% confidence interval (CI) 1.16–2.17) [14], and the lowest odds were attributed to offspring of primigravid placenta infected mothers.

Regarding the early appearance of parasites in infants, the above mentioned study in Tanzania reported a 1.41 estimated hazard ratio (HR) (95% CI 1.01–1.99) of first parasitaemia for offspring of mothers with *P. falciparum* placental infection after adjusting for gravidity, transmission season at time of birth, area of residence, and bed net usage [14]. In Gabon, a significant correlation between placental malaria and the first malaria episode was also found (adjusted HR (AHR) = 2.1; 95% CI 1.2–3.7) after adjustment for gravidity, season of birth, area of residence, IPTp versus placebo, and ITNs [15]. A more recent study in Mozambique found that infants born to women who had clinical malaria during pregnancy, or acute placental infection, had an increased risk of clinical malaria during infancy (OR = 1.96; 95% CI, 1.13–3.41, and OR = 4.63; 95% CI 2.10–10.24, respectively) [22]. Furthermore, a cohort study conducted in Tori Bossito (Benin) confirmed the link between PM and malaria in infants through consistent entomologic and environmental follow-up [17,23]. The study findings on infants sleeping in a house with an ITN confirmed the link between PM and malaria controlled for transmission intensity, seasonality, number of anopheles, antenatal care (ANC) visits, and maternal severe anaemia (AHR = 2.13; 95% CI 1.24–3.67) compared with infants whose mothers did not have placental malaria at delivery. This cohort study additionally reports an increased susceptibility of infants to *P. falciparum* parasites with antigens to which they were previously exposed *in utero* suggesting an immune tolerance process undergoing during pregnancy [32]. PAM has also been associated with a reduction in maternal antibody transfer to the foetus [33,34], hence increasing infant susceptibility to parasites [35,36]. Consistent with the notion that the type, timing, and the duration of exposure to the parasite *in utero* determine susceptibility to malaria, infections occurring during the third trimester are associated with increased risk of infection and clinical malaria during the first year of life according to another study in the province of Mono (South Benin) [24].

Nevertheless, the effect of PAM on infant health may involve an overall increased morbidity and mortality. Placental malaria was additionally correlated with non-malaria infections in the Tori Bossito cohort infants during the first 18 months of life suggesting that immune tolerance could also imply immunity in a more general manner besides malaria specific immunity [20]. Moreover, placental malaria posed a significant risk factor for overall mortality during the first year of life [37] in a study in Malawi, and another study from Mozambique [22] identified both acute placental malaria and cord blood parasitaemia with increased infant mortality. More precisely, in this study from Mozambique infant mortality was also significantly associated with malaria infection of the placenta (p-value < 0.012) after adjustment on HIV status, LBW, maternal clinical malaria during pregnancy, foetal anaemia and IPTp regime. The risk of dying during infancy was increased among infants born to women with acute placental infection (OR = 5.08; 95% CI 1.77–14.53), as well as among infants with parasitaemia in the cord blood (OR = 19.31; 95% CI, 4.44–84.02).

A possible explanation for different immune tolerance effects of PAM relates to HLA-G polymorphisms and their association with different malaria susceptibility [38]. HIV infection influences as well a woman’s susceptibility to malaria, and this is of major concern as both diseases overlap considerably in sub-Saharan Africa. Consistent evidence suggests both infections interact synergistically and result in poorer health outcomes [39]. PAM is more frequent among HIV infected women in comparison to non-infected women, and can increase maternal HIV load [40-42]. PAM in HIV-positive pregnant women is further associated with higher risk of both anaemia and LBW [40,43-45]. This results in overall increased maternal and infant mortality [46,47].

A potential long-term consequence of PAM concerns neuro-cognitive impairment of infants exposed to malaria *in utero*. Due to recent evidence concerning the role of the complement system in the regulation of neurodevelopment, it has been proposed that excessive complement activation induced by placental malaria may disrupt normal neurodevelopment resulting in neurocognitive impairment of infants exposed to *Plasmodium in utero*[48].

Although a complete explanation of the physiopathology of PAM has not yet been understood, *in utero* exposure to malaria is probably nonetheless correlated with placental sequestration of erythrocytes. The immune tolerance process would plausibly then depend on the type of malaria antigen in contact with the foetus, the amount and the duration of the exposure, and the timing of exposure during pregnancy [16,49]. The interaction between gestation and infection timing during pregnancy has been previously shown to influence the pathologic consequences for the offspring. Due to the particular physiopathology of PAM, a specific immunity develops during the first pregnancy [10] and, hence, primigravidae are at higher risk of PAM compared to multigravidae [10]. In this respect, infants of primigravid women are also at higher risk of subsequent malaria in comparison to infants of multigravid women, mainly as a result of reduced antibody transfer [11]. Finally the timing of malaria episodes during pregnancy results in different effects on both the mother and the foetus; parasitaemia appears to be higher during the first and second trimesters, even if follow-up on *P. falciparum* parasitaemia during the first trimester has seldom been complete [10,50-53]. Essentially, the administration of IPTp at different moments determines different protection patterns for the infant [50] and, in parallel, a significant reduction in placental malaria and maternal parasitaemia has been extensively described [54] following the implementation of PAM control interventions. As a result of the different infant malaria outcomes depending on PAM and IPTp, and considering the body of the available research, the following questions are posed: How does exposure *in utero* to *P. falciparum* influence malaria in infants? How do control interventions modify in turn the impact of PAM on clinical malaria in infants?

### Pregnancy associated malaria and control interventions: effect on perinatal malaria outcomes

**Figure 1:**
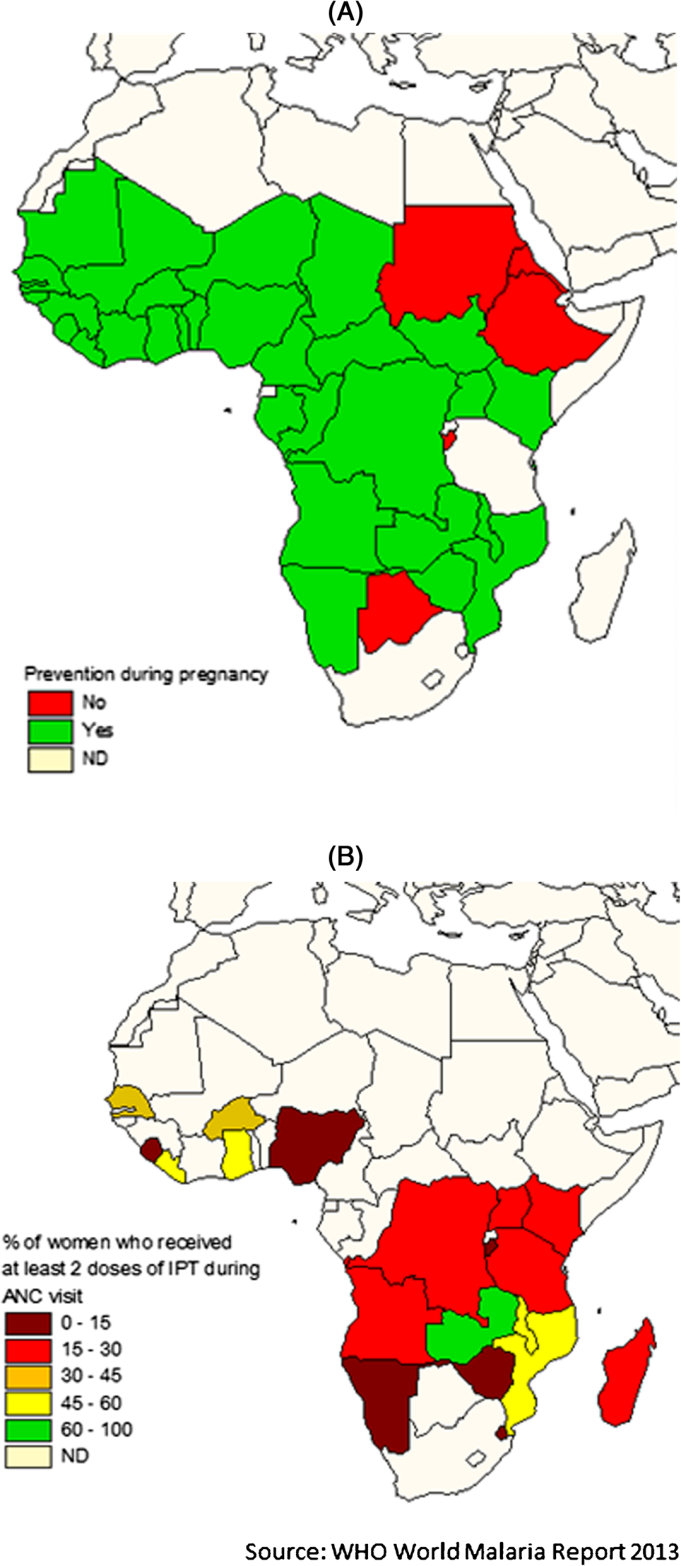
**Intermittent Preventive Treatment in pregnancy in Africa.** Source: World Malaria report 2013. WHO publications 2013. **A**. Implementation of intermittent preventive treatment in pregnancy in Africa. **B**. The percentages of women having received at least 2 doses of IPTp are approximated data issue of the latest demographic surveys of the countries represented.

Foetal exposure to *Plasmodium in utero* primarily depends on transmission and control interventions. Preventive measures substantially alter the interaction between exposure and immunity. IPT is a widespread preventive strategy to fight malaria and involves the administration of a curative dose of an effective anti-malarial drug, regardless of the presence of *Plasmodium* in the blood, to prevent the disease [19]. IPT measures decrease parasitaemia, and consequently influence the immunity response of the infant to *Plasmodium in utero* through maternal intermittent preventive treatment in pregnancy (IPTp). Therefore, WHO recommends IPTp with SP for all pregnant women as early as possible in the second trimester, and at each scheduled antenatal care visit at least one month apart in areas of moderate to high malaria transmission [55], IPTp strategies are however not yet completely deployed in malaria endemic regions and the implementation of IPTp interventions interfere with PAM outcomes. Figure 1 presents the main characteristics concerning implementation of IPTp programmes in Africa.

Effective administration of IPTp clears placental parasitaemia and consequently modifies the exposure to malaria antigens resulting in a significant reduction in placental malaria and maternal parasitaemia [54]. Compared to case management or placebo in pregnant women, a two-dose IPTp regime with sulphadoxine-pyrimethamine (SP) significantly reduced placental malaria according to a review on four studies (relative risk (RR) = 0.48) [56]. In a randomized, double blind, placebo-controlled trial with joint use of ITNs in Mozambique, SP-IPTp (1-2 doses) was correlated to a significant decrease only in active placental malaria [57] (Table 1). In Mali, placental parasitaemia was significantly reduced by SP-IPTp (AOR = 0.69) when compared to weekly administered chloroquine (CQ) [58] and confirmed higher SP efficacy compared to CQ already reported in Malawi [59]. A recent meta-analysis has concluded significant reduction in PM after three doses of SP compared to two doses [54], which corresponds to current WHO recommendations.

A comprehensive review encompassing published studies conducted between 1985 and 2000 found a PAM prevalence range of 10% to 65% among all gravidae [12], with a median prevalence of 27.8% [10]. In low-transmission African settings, the median prevalence peripheral infection was 13.7% and the placental malaria median prevalence was 6.7% [10]. Recent studies however reported a significant decline in prevalence following PAM control interventions. The protection of joint ITNs with IPTp-SP use is significant in only certain trials, yet reported ITN use ranges from 5 to 25%, and this might not be sufficient enough to show an effect [60]. An article reviewed the influence of preventive measures on PAM during a decade, effectively 2002 to 2012, and reported placental malaria rates ranging from 2 to 29% among women treated with less than three doses (mainly two) of sulphadoxine-pyrimethamine (SP) compared to 2 to 8% among women receiving more than or equal three doses (mainly three) [60]. A novel study included within the afore mentioned review describes a two-fold lower prevalence of placental malaria in the three-dose SP group compared to the two-dose SP group (adjusted prevalence ratio = 0.48) [61]. Even if the augmented efficacy associated with higher doses is predominately observed in clinical trials rather than in studies of public health programme implementations [60], the emergence of SP resistance is certainly shaping the efficacy of IPTp, and consequently its influence on the malaria burden in infants.

### IPTp and malaria in infants: when protection encounters resistance

Reduced compliance with drug regimes and the increasing resistance to anti-malaria drugs highlight the complexity of IPTp management at present. A 2007 meta-analysis confirmed that SP IPTp continued to benefit pregnant women in areas of up to 39% resistance to SP, measured by *in vivo* resistance at day 14 of treatment in children [56]. Similar results were found in Benin, where rates of *in vivo* resistance to SP were estimated to be 50% by day 28 of treatment in infants, and yet SP IPTp succeeded to prevent LBW [62]. However, studies published more recently display contradictory results. A study in Malawi, where there is a strong fixation of the resistant quintuple mutant, shows significantly reduced small for gestational age (SGA) rates in offspring of primigravid women having received ≥2 doses of SP compared to 0-1 doses [63]. On the other hand, peripheral parasitaemia was is significantly higher among women having received ≥2 doses of SP. Indeed, the effects of resistance on malaria clinical outcomes become more frequent in more recent studies from East Africa. In a Tanzanian site with high SP resistance (14-day parasitologic SP treatment failure rate in children of 68%), IPTp was not associated with a reduction in odds of PM, LBW or maternal anaemia. Furthermore, it was associated with increased odds of foetal anaemia and severe malaria among the offspring (AOR = 2.31) [64]. IPTp in this setting was associated with an overall increased risk of severe malaria [64,65].

However a recent longitudinal study revealed no significant increase of malaria at delivery after IPTp treatment, albeit the increasing prevalence and fixation of SP-resistant *P. falciparum* haplotypes in another area in Malawi [66]. Evidence for the present efficacy of SP-IPTp regimes is inconclusive but resistance to SP is spreading. Close monitoring of its efficacy is therefore necessary to determine if or when the treatment failure of SP-IPTp detected by some recent studies has become generalized at the population level, thus necessitating a switch to alternative drug regimes. Nevertheless, the Malaria Policy Advisory Committee (MPAC) cited a current paucity of data in order to determine the precise level of resistance obliging interruption of IPTp-SP treatment, especially in the absence of an established and effective alternative [55].

Currently, the Intermittent Screening and Treatment (IST) is a proposed alternative to IPTp in areas with substantial resistance against IPTp regimes. IST consists in screening for malaria infection using a malaria rapid diagnostic test (RDT) at scheduled antenatal clinic visits and subsequently treating positive women with an effective anti-malarial drug [67]. However, extensive evidence on IST efficacy is lacking in African regions and further efficacy studies should be conducted in broader geographical regions [68].

In summary, PAM determines foetal exposure to *P. falciparum in utero* and is hence correlated to congenital malaria and earlier development of clinical episodes in infancy, possibly as the consequence of an immune tolerance process *in utero*. Effective IPTp diminishes PM and malaria associated morbidity such as LBW, pre-term delivery, IUGR, and perinatal mortality in areas where resistance to SP is not highly significant. Yet the influence of different IPTp regimes on malaria morbidity in infants remains a question for further research. The concrete effect of resistance and the ongoing immune tolerance process *in utero* have not been presently explored. Further evidence is also lacking on the importance of the timing of infection during pregnancy and infant malaria morbidity. There exists some evidence that earlier administration of IPTp has a positive effect on birth outcomes like LBW, nevertheless, later dosing provides a more continuous protection [50], thus necessitating the administration of three doses instead of two for improved clinical outcomes. In addition, the implementation of different IPTp regimes should be adapted according to transmission and the SP-resistance pattern. For example, IST has been applied successfully in an area of moderately high malaria transmission in Ghana [67]. IST should be further explored and its efficacy should be evaluated in other transmission settings to ascertain its utility as an effective tool for the control of PAM.

## Conclusions

This review on the impact of PAM on malaria in infants substantiates the complexity of the subject and the necessity of a holistic approach for fighting malaria. In addition, research gaps should be fulfilled to enhance malaria outcomes. Strategies should start during the pre-conceptual period or at least during pregnancy, as there is evidence of increased infant susceptibility to parasites carrying antigens to which they were previously exposed while *in utero*. A complete explanation of the immune process remains a question for further research as well as the precise effect of the timing of *in utero* exposure to the parasite. Furthermore, the role of protective maternal antibodies has not yet been clarified. Operational research on different preventive IPT strategies should also be continuously conducted, and cost effectiveness analysis for community-level IST interventions should be investigated.

Finally, novel aspects of research on PAM should be further explored. Due to the long-term impact of placental malaria’s possible neuro-cognitive consequences, the scientific community should prioritize studies investigating this interaction. An exploration of the influence of HLA-G polymorphisms on subsequent malaria symptoms would serve as well as an important contribution for infant malaria risk factors.

## Abbreviations

ACT: Artemisinin-based combination therapy; AHR: Adjusted hazard ratio; AL: Artemether-lumefantrine; ANC: Antenatal care; AOR: Adjusted odds ratio; AQ: Amodiaquine; CQ: Chloroquine; HR: Hazard ratio; IPTp: Intermittent preventive treatment in pregnancy; IST: Intermittent Screening and Treatment; ITNs: Insecticide-treated nets; IUGR: Intra-uterine growth retardation; LBW: Low birth weight; MeSH: Medical Subjects Headings; MPAC: Malaria Policy Advisory Committee; MQ: Mefloquine; OR: Odds ratio; PAM: Pregnancy associated malaria; PM: Placental malaria; RDT: Rapid diagnostic test; RR: Relative risk; SGA: Small for gestational age; SP: Sulphadoxine-pyrimethamine; SPR: Slide positivity rate.

## Competing interests

The authors declare that they have no competing interests.

## Authors’ contributions

VMA gathered and selected the articles, realized the article database and drafted the manuscript. RA realized the figure and helped to draft the manuscript. MC participated in the design and coordination of the article and helped to draft the manuscript. All authors read and approved the final manuscript.
